# Relationship between risk markers for cardiovascular disease and peri-implant diseases

**DOI:** 10.1186/s40729-020-00273-z

**Published:** 2020-11-25

**Authors:** Gülbahar Ustaoğlu, Emrah Erdal

**Affiliations:** 1 Dentistry Faculty, Department of Periodontology, Bolu Abant İzzet Baysal University, 14300 Bolu, Turkey; 2 Medical Faculty, Department of Cardiology, Bolu Abant İzzet Baysal University, Bolu, Turkey

**Keywords:** Peri-implantitis, Risk factors, Triglycerides, Uric acid, Vitamin D

## Abstract

**Background:**

The aim of this paper is to explore the serum biochemical parameters also known as risk markers for cardiovascular system, in individuals who have received dental implant treatment, and to reveal risk factors for peri-implant diseases.

**Methods:**

The study included 58 subjects with peri-implantitis, 49 subjects with peri-implant mucositis, and 49 healthy subjects. All the subjects were assessed in terms of both peri-implant clinical parameters—probing depth (PD), bleeding on probing (BOP), the type of prosthesis, gingival index (GI), keratinized mucosa width (KMW), and plaque index (PI)—and serum biochemical parameters (e.g., LDL cholesterol, HDL cholesterol, triglyceride, total cholesterol, vitamin D, uric acid, white blood cell (WBC), neutrophil, hemoglobin (Hb), and platelet counts).

**Results:**

KMW was the lowest in the peri-implantitis group. Compared with the other groups, the peri-implantitis group showed significantly higher levels of triglyceride, uric acid, and WBC. The peri-implantitis group had the lowest level of vitamin D. Triglyceride and uric acid levels had positive correlations with peri-implant clinical parameters.

**Conclusion:**

High levels of triglyceride and uric acid may pose a risk for both peri-implant diseases and cardiovascular diseases. Prior to dental implant surgery, patients’ serum biochemical parameters should be checked.

## Introduction

Currently, dental implants have many applications in the treatment of patients with either partial or complete edentulism. Despite the success of implants and their high survival rate, the prevalence of peri-implant diseases has increased [1]. Infectious peri-implant diseases are classified in two groups, namely, peri-implant mucositis and peri-implantitis. While the former (also known as implant gingivitis) can be defined as a reversible inflammation of the soft tissues embracing an implant, the latter (i.e., periodontitis) is an inflammation leading to loss of supporting bone around an implant [2]. No predictable approach is currently available for treating peri-implant diseases. Hence, preventive measures before the occurrence of any peri-implant disease become crucial. Accordingly, the first steps to establish successful strategies for prevention involve the identification of risk factors [3]. A number of risk factors/indicators may directly increase the likelihood of peri-implant mucositis and peri-implantitis [4]. Potential risk factors for developing peri-implant diseases include smoking, a history of periodontitis, poor oral hygiene, diabetes mellitus, alcohol consumption, genetics, and related factors for prosthetic and implant surface characteristics [5, 6].

Over the years, some papers have focused on the coexistence of medical conditions with peri-implantitis [7–9]. Evidence suggests that bone tissues may alter as a result of high levels of triglycerides and cholesterol as well as cardiovascular diseases. An excessive level of low-density lipoprotein cholesterol (i.e., dyslipidemia) contributes to a poor bone metabolism or osseointegration of dental implants [10]. The mechanisms of the relationship between hyperlipidemia and bone tissue metabolism are related aspects of several metabolic changes, including low bone mineral density, increased osteoclast count, and inhibition of osteoblastic activity [11, 12]. Also, the levels of statins, vitamin D, and cholesterol are closely inter-correlated as well. Interestingly, it is worth to note that 7-dehydrocholesterol is the same precursor of both vitamin D and cholesterol [13]. As far as the bone is concerned, vitamin D has an activating effect on osteoclasts, increasing the amount of extracellular matrix proteins synthesized by osteoblasts [14]. There are several studies in the literature reporting that hypertension, dyslipidemia with high values of LDL and triglyceride, low values of HDL, and vitamin D may pose a risk for peri-implantitis [15–17]. Furthermore, altered levels of circulatory lipoproteins with decreased HDL, and increased LDL and triglyceride, indicate a higher risk of atherosclerotic cardiovascular diseases [18]. Also cross-sectional studies have reported that vitamin D deficiency is associated with increased risk of CVD and mortality [19, 20]. Elevations of serum lipids and low serum levels of 25-hydroxyvitamin D may have a serious negative impact on periodontal and cardiovascular health [21–24].

Thus, it has been reported that elevated serum uric acid levels increase oxidative stress and inflammation [25, 26]. Several studies showed an association between elevated serum uric acid levels and some disorders such as hypertension, atherosclerosis, renal disease, obesity, insulin resistance, and dyslipidemia [27]. To the best of our knowledge, there is no study in the literature about uric acid levels in patients with peri-implant diseases.

The aim of the present study was to investigate the serum biochemical parameters which are cardiovascular disease risk markers in individuals who have received dental implant treatment and to reveal risk factors for peri-implant diseases.

## Materials and methods

The approval was obtained for this cross-sectional study from the Clinical Research Ethics Committee of the Bolu Abant İzzet Baysal University. The study was conducted in compliance with the ethical principles according to the Declaration of Helsinki.

The study included 58 subjects with peri-implantitis, 49 subjects with peri-implant mucositis, and 49 healthy subjects, all of whom comprised patients having visited the Department of Periodontology at the Bolu Abant İzzet Baysal University. All participants signed an informed consent.

Diagnosis of peri-implant diseases was performed in compliance with the 2017 World Workshop on the Classification of Periodontal and Peri-Implant Diseases and Conditions [28].

The diagnostic definition of peri-implant health was based on the following criteria: the absence of clinical signs of inflammation, bleeding, and/or suppuration during gentle probing; and the absence of bone loss on radiography. Criteria for selecting the patients with peri-implant mucositis were as follows: the presence of bleeding and/or suppuration during gentle probing and the absence of bone loss on radiography. Criteria for selecting the patients with peri-implantitis were as follows: the presence of bleeding and/or suppuration during gentle probing and radiographic bone levels of at least 3 mm apical of the most coronal fragment of the intra-osseous part of the implant. The subjects in the study population were required to have at least one dental implant in use for 36 months. The exclusion criteria were as follows: systemic administration of antibiotics (or prophylactic antibiotics) for the last 3 months, pregnancy or breast feeding, having diabetes mellitus, and a history of malignancy, radiotherapy, chemotherapy, or immunodeficiency within the last 4 years.

### Clinical and radiographic evaluation of peri-implants

All clinical and radiographic examinations were performed by the same person using a typical periodontal probe graded in millimeters (Williams: Hu-Friedy, Chicago, IL, USA). The patients were monitored, performing clinical and radiographic examinations on a regular basis. Digital periapical radiographic images were obtained to reveal any threats for diagnostic purposes only.

Records of clinical parameters for the peri-implant were as follows: probing depth (PD) at 6 sites per teeth and implant (the distance between the mucosal margin and the probeable sulcus/pocket), gingival index (GI), plaque index (PI), either the presence (1) or absence (0) of bleeding on probing (BOP) at six sites for each implant, and keratinized mucosa width (KMW).

### Calibration process conducted by the researcher

The researcher carried out a calibration process in 10 peri-implantitis patients who were not enrolled in the study. Evaluation of the probing pocket depth was performed at intervals of at least 60 min, and the overall value of kappa to calculate the reliability within each examiner was 0.89, representing an acceptable degree of agreement.

### Medical examination

Blood samples from the participants were collected after a 12-h period of fasting. Subsequently, we evaluated the levels of HDL and LDL cholesterols, total cholesterol, triglyceride, vitamin D, creatine, urea, and uric acid. We also analyzed the key components in the complete blood count, such as WBC, neutrophil, Hb, mean corpuscular volume (MCV), platelet counts, mean platelet volume (MPV), and plateletcrit (PCT).

### Statistical analysis

All analyses were performed using SPSS 18.0 Statistical Package Software for Windows OS (SPSS Inc., Chicago, Illinois, USA). Power calculations demonstrated that a minimum of 30 samples per group was required to compare data at *α* = 0.05 with a power value of 85%.

Quantitative variables are expressed as mean ± standard deviation (SD) and median (interquartile range (IQR)). To assess the differences between these groups, we used one-way ANOVA analysis with Bonferroni correction for normally distributed variables, Kruskal Wallis test for variables without normal distribution, and chi-squared test for qualitative variables. ANCOVA was used to compare the three groups in terms of biochemical parameters by adjusting for ages.

The correlations of triglyceride, vitamin D, uric acid, GI, PD, and BOP were assessed using Spearman’s correlation analyses. All the results were deemed statistically significant at the *p* ≤ 0.05 level.

## Results

A total of 156 patients were recruited in the study. The distribution of patients’ age, gender, medical history, implant number, implant position, prosthesis type, and antagonists peri-implant parameters among groups are shown in Table 1. The frequency of smoking and alcohol was not different between the groups (Table 1). The smallest KMW was observed in the peri-implantitis group (*p* < 0.001).

**Table 1:** Characteristics of the study groups

	Peri-implantitis group (***N*** = 58 )	Peri-implant mucositis group (***N*** = 49 )	Healthy implant group (***N*** = 49)	***P*** value
Age (median (IQR)	55 (17.5)	53 (17.7)	50 (18.5)	0.181
Gender (female/male)	33/25	27/21	33/15	
Smoke (%)	7 (12)	4 (8.1)	1 (2.0)	0.271
Alcohol (%)	1 (1.7)	0 (0)	0 (0)	0.506
PD (median (IQR)	5 (1.6)	3 (1.0)	2.5 (1.0)	**< 0.001**
PI (median (IQR)	2 (1.0)	2 (0.8)	0 (0)	**< 0.001**
GI (median (IQR)	2.0 (0.1)	2.0	0	**< 0.001**
BOP (%)	77.6	100	0	**< 0.001**
KMW (median (IQR)	1.5 (1.0)	2.0 (1.0)	2.5 (1.0)	**< 0.001**
Number of implants	81	55	60	
Implant position (maxilla/mandible)	39/42	18/37	28/32	
Prosthesis type (%)				
Multiple unit	38 (46.9)	20 (36.3)	13 (21.6)	
Single crown	19 (23.4)	23 (41.8)	41 (68.3)	
Overdenture	24 (29.6)	12 (21.8)	6 (10)	
Antagonists (%)				
Natural teeth	29 (35.8)	25 (45.4)	34 (56.6)	
Bridge restoration	20 (24.6)	10 (18.1)	10 (16.6)	
Overdenture	5 (6.1)	1 (1.8)	2 (3.3)	
Removable denture	9 (11.1)	13 (23.6)	6 (10)	
Implant supported prosthetic restoration	18 (22.2)	6 (10.9)	8 (13.3)	

*N* number of participants, *PD* probing depth, *PI* plaque index, *GI* gingival index, *BOP* bleeding on probing, *KMW* keratinized mucosa width, *IQR* interquartile range

Compared with the other groups, the peri-implantitis group showed significantly higher levels of triglyceride and uric acid (Table 2). After age adjustment, the PCT value was significantly higher in the peri-implantitis group (Table 2). The vitamin D value was found the lowest in the peri-implantitis group. WBC value was statistically high in peri-implantitis group, and no significant difference was found between the other parameters in the complete blood count (Table 2). There was a positive correlation between triglyceride values and clinical parameters. Also, there was a positive correlation between uric acid and GI, PD, BOP, KMW values (*r* = 0.238, *p* = 0.006; *r* = 0.464, *p* ≤ 0.001; *r* = 0.230, *p* = 0.008; *r* = − 0.240, *p* = 0.006, respectively) (Table 3). Vitamin D had a negative correlation with GI values (*r* = − 0.191, *p* = 0.020). There was no significant correlation between PCT values and clinical parameters (Table 3).

**Table 2:** Biochemical parameters in the study groups

	Peri-implantitis (***N*** = 58 )	Peri-implant mucositis (***N*** = 49 )	Healthy implant (***N*** = 49 )	***P*** value	***P*** value (age-adjusted)
LDL-C (mean ± Sd)	130.2 ± 31.4	119.5 ± 38.5	120.1 ± 37.5	0.498	0.674
HDL-C (median (IQR)	47 (20.5)	52 (27.2)	54 (19)	0.063	0.081
Triglyceride (median (IQR)	148 (107.5)^a^	125 (93)^ab^	95 (61)^b^	**< 0.001**	**< 0.001**
TOTAL-C (mean ± Sd)	213.4 ± 39.1	198.6 ± 44.3	202 ± 41.3	0.145	0.221
Vitamin-D (median (IQR)	13.8 (8.9)^a^	15.5 (11.9)^ab^	17.9 (12.7)^b^	**0.044**	**0.043**
Uric acid (median (IQR)	5.1 (2.1)^a^	4.4 (1.5)^b^	4.0 (1.4)^b^	**< 0.001**	**< 0.001**
Creatine (median (IQR)	0.84 (0.2)	0.86 (0.1)	0.77 (0.1)	0.090	0.476
WBC (mean ± Sd)	7.5 ± 1.9^a^	6.6 ± 1.6^ab^	6.4 ± 1.1^b^	**0.030**	**0.036**
Neutrophil (mean ± Sd)	4.0 ± 1.4	4.0 ± 1.7	3.9 ± 1.0	0.848	0.887
Hemoglobin (median (IQR)	13.7 (2.0)	14 (1.8)	13.9 (1.6)	0.673	0.755
MCV (median (IQR)	86 (73)	86 (6.2)	87 (5.5)	0.689	0.631
Platelets (median (IQR)	246 (75)	227 (62)	244 (54)	0.160	0.169
MPV (mean ± Sd)	8.5 ± 1.1	8.7 ± 1.0	8.6 ± 1.3	0.850	0.930
Plateletcrit (median (IQR)	0.211 (0.07)^a^	0.202 (0.05)^b^	0.209 (0.04)^ab^	0.109	**0.044**

*N* number of participants, *LDL-C* low-density lipoprotein cholesterol, *HDL-C* high-density lipoprotein cholesterol, *TOTAL-C* total cholesterol, *WBC* white blood cell, *MCV* mean corpuscular volume, *MPV* mean platelet volume, *Sd* standard deviation, *IQR* interquartile range

If the any group mean carries superscript different letters (a, b, c) from the other group mean, it indicates that the difference between them is statistically significant difference

**Table 3:** Spearman correlation analysis among biochemical and peri-implant parameters

	GI	PD	BOP	KMW
Uric acid	*r* = 0.238, *p* = **0.006**	*r* = 0.464, *p* = **< 0.001**	*r* = 0.230, *p* = **0.008**	*r* = − 0.240 *p* = **0.006**
Vitamin D	*r* = - 0.191, *p* = **0.020**	*r* = 0.060, *p* = 0.463	*r* = - 0.137, *p* = 0.093	*r* = 0.040 *p* = 0.627
Triglyceride	*r* = 0.263, *p* = **0.001**	*r* = 0.331, *p* = **< 0.001**	*r* = 0.224, *p* = **0.006**	*r* = − 0.216 *p* = **0.010**
PCT	*r* = 0.048, *p* = 0.555	*r* = 0.109, *p* = 0.177	*r* = 0.116, *p* = 0.153	*r* = − 0.141 *p* = 0.085

*GI* gingival index, *PD* probing depth, *BOP* bleeding on probing, *KMW* keratinized mucosa width, *PCT* plateletcrit

## Discussion

Latest studies have revealed that lipid metabolism may be changed by chronic local and acute systemic infections which are involved in the plasma concentrations of unregulated cytokines such as TNF-α and IL-1β [29–31]. Thus, through action of TNF-α and IL-1β, exposure to microorganisms/endotoxin results in elevated levels of free fatty acids, LDL, and triglyceride [32]. Also, these cytokines play a major role in periodontal destruction too [33, 34]. Recently, some researchers have suggested that hypercholesterolemia may be associated with dental implant osseointegration [35, 36]. It has been hypothesized that there is a relationship between hypercholesterolemia and a higher failure rate of implants and bone grafts, and the authors recommend preventive evaluation of cholesterolemia concentration before implant surgery [10]. It is also known that high LDL and triglyceride values and low HDL are important risk factors for coronary artery disease [37, 38]. Although some studies reported that a history of cardiovascular diseases was associated with peri-implantitis [39, 40], the relationship between peri-implantitis and cardiovascular diseases was not clearly demonstrated. In this study, we investigated the serum biochemical parameters which are cardiovascular disease risk markers in patients with dental implants and possible risk factors in patients with peri-implant diseases.

According to our results, no statistically significant difference was found between the groups in terms of LDL-C, HDL-C, and TOTAL-C values; however, the peri-implantitis group had higher levels of triglyceride and uric acid. There was a positive correlation between uric acid, triglyceride, and GI, PD, BOP, and KMW values in our study.

Peri-implantitis and cardiovascular disease may share common risk factors, and association between peri-implantitis and coronary heart disease may be due to the elevated levels of plasma lipids. In a study of Vohra et al. [41], they found a correlation between higher total cholesterol and triglyceride levels and increased risk of peri-implant disease. Alasqah et al. [42] reported that peri-implant indices including PI, BOP, crestal bone loss, and triglycerides as well as levels of low-density lipoprotein and total cholesterol were significantly higher in patients with obesity compared with non-obese subjects. Similarly, another study showed a statistically significant difference in marginal bone loss between patients with and without obesity even following an adjustment of metabolic parameters such as total cholesterol and triglyceride [43].

There are several studies which report significant association between plasma lipid levels and the severity of the periodontal disease [44, 45]. In our study, there was a positive correlation between triglyceride values and GI, PD, and BOP values. These results can be explained by the bidirectional relationship between hyperlipidemia and peri-implantitis. Thus, Lachmann et al. [40] has shown a significant comorbidity in individuals with cardiovascular diseases and evidence of implants and with a high prevalence of moderate plaque and BOP. Another study concluded that odds ratio of having peri-implantitis and cardiovascular disease was 8.7 after adjusting for age, smoking status, and gender [39].

Aside from the positive effects of vitamin D on osseointegration in some preclinical studies [46–48], it remains unclear whether vitamin D supplementation can effectively accelerate peri-implant bone healing. In our study, vitamin D values were found lowest in peri-implantitis group and also were lower in peri-implant mucositis group than healthy implant group. A retrospective study by Mangano et al. [49] evaluated correlation between early implant failure and low serum levels of vitamin D and showed a higher incidence of the implant failure rate in these patients but a correlation between both factors could not be determined. Furthermore, Acipinar et al. [17] evaluated fibroblast growth factor (FGF)-23 and 25-hydroxy-vitamin D_3_ (25(OH) D3) levels in peri-implant sulcus fluid in peri-implant health and diseases. They found that the total amount of 25(OH) D3 was significantly lower in the peri-implantitis group compared with the peri-implant healthy subjects.

In many studies, uric acid has been shown to play an important role in the occurrence and development of coronary artery disease [50–52]. Furthermore, uric acid could stimulate inflammatory response [53]. Production of proinflammatory cytokines, such as interleukin-1β (IL-1β), IL-6, and tumor necrosis factor-α (TNF-α), in human mononuclear cells are stimulated by uric acid [54]. We found that uric acid levels were significantly higher in the peri-implantitis group compared with the other groups. Also, there was a positive correlation between uric acid levels and GI, PD, BOB, and KMW values. Our results support that elevated serum uric acid level may be associated with inflammation too.

After age adjustment, we found that PCT value was significantly higher in the peri-implantitis group, but no significant correlation was found between the PCT values and clinical parameters. In a study by Ustaoğlu et al. [55], PCT and MPV values were found significantly higher in patients with periodontitis, and there was a statistically significant relationship between PCT values and clinical parameters. They concluded that these markers might be a useful marker to determine an increased thrombotic state and inflammatory response in periodontal diseases. Peri-implantitis may also stimulate infectious and immune response and cause the development of atherogenesis, coronary heart disease, and myocardial infarction.

Although previous studies showed a strong association between periodontitis and systemic diseases including cardiovascular diseases and diabetes, the relationship between peri-implantitis and cardiovascular diseases was not clearly demonstrated [56, 57]. Thus, some studies concluded that no statistically significant association between cardiovascular diseases and peri-implantitis were observed [8, 58, 59].

In our study, there were some limitations; coronary angiography was not performed to evaluate coronary artery disease, and radiographic measurement was not done to calculate amount of crestal bone loss around dental implants. Furthermore, the analyses of peri-implant crevicular fluid in patients for the purpose of detecting proinflammatory cytokines may reveal any local destruction around dental implants. The limited sample size is another limitation of this study. Further research with a larger sample size is required to confirm these observations.

## Conclusion

In conclusion, we found significantly higher levels of triglyceride and uric acid (i.e., risk markers for a cardiovascular disease) in peri-implantitis group compared with other groups. Long-term clinical trials with larger sample size are required to confirm the relationship between these risk markers and cardiovascular and peri-implant diseases.

## Data Availability

The datasets used and/or analyzed during the current study are available from the corresponding author on reasonable request.

## Abbreviations

PD: Probing depth
BOP: Bleeding on probing
GI: Gingival index
KMW: Keratinized mucosa width
PI: Plaque index
WBC: White blood cell
Hb: Hemoglobin
MCV: Mean corpuscular volume
MPV: Mean platelet volume
PCT: Plateletcrit
